# Update: Gender differences in CABG outcomes—Have we bridged the gap?

**DOI:** 10.1371/journal.pone.0255170

**Published:** 2021-09-15

**Authors:** Robina Matyal, Nada Qaisar Qureshi, Syed Hamza Mufarrih, Aidan Sharkey, Ruma Bose, Louis M. Chu, David C. Liu, Venkatachalam Senthilnathan, Feroze Mahmood, Kamal R. Khabbaz

**Affiliations:** 1 Department of Anesthesia, Critical Care and Pain Medicine, Beth Israel Deaconess Medical Centre, Harvard Medical School, Boston, MA, United States of America; 2 Division of Cardiac Surgery, Department of Surgery, Beth Israel Deaconess Medical Center, Harvard Medical School, Boston, MA, United States of America; Case Western Reserve University School of Medicine, UNITED STATES

## Abstract

**Background:**

Appreciation of unique presentation, patterns and underlying pathophysiology of coronary artery disease in women has driven gender based risk stratification and risk reduction efforts over the last decade. Data regarding whether these advances have resulted in unequivocal improvements in outcomes of CABG in women is conflicting. The objective of our study was to assess gender differences in post-operative outcomes following CABG.

**Methods:**

Retrospective analyses of institutional data housed in the Society of Thoracic Surgeons (STS) database for patients undergoing CABG between 2002 and 2020 were conducted. Multivariable regression analysis was conducted to investigate gender differences in post-operative outcomes. P-values were adjusted using Bonferroni correction to reduce type-I errors.

**Results:**

Our final cohort of 6,250 patients had fewer women than men (1,339 vs. 4,911). more women were diabetic (52.0% vs. 41.2%, p<0.001) and hypertensive (89.1% vs. 84.0%, p<0.001). Women had higher adjusted odds of developing ventilator dependence >48 hours (OR: 1.65 [1.21, 2.45], p = 0.002) and cardiac readmissions (OR: 1.56 [1.27, 2.30], p = 0.003). After adjustment for comorbidity burden, mortality rates in women were comparable to those of age-matched men.

**Conclusion:**

The findings of our study indicate that despite apparent reduction of differences in mortality, the burden of postoperative morbidity is still high among women.

## Introduction

Coronary artery bypass graft (CABG) surgery remains the preferred option for advanced cardiovascular disease [1]. Despite the introduction of new techniques and procedural innovations, improvements in outcomes of CABG surgery have not been uniform across genders [2]. This observation has been attributed to gender-based anatomical and physiological factors such as smaller coronary artery size [3], heart failure with preserved ejection fraction, underutilization of internal mammary artery (IMA) for CABG [4] and role of post-menopausal estrogen withdrawal in atherosclerosis [5]. Delayed onset and ambiguity of symptoms of coronary artery disease (CAD) leading to delayed diagnosis has also been implicated [6]. Epidemiological and clinical studies in cardiovascular risk stratification are either gender neutral or women are under-represented. Recent appreciation and awareness of these factors in the public and medical community has led to a paradigm shift in the management of women with CAD [6, 7]. These initiatives are targeted to establish gender parity in utilization of diagnostic tests, cardioprotective and reperfusion therapies [6, 7]. Furthermore, bench research is being conducted to understand the molecular basis of these differences and explore targeted therapies [5]. As result of these initiatives, almost a 30% reduction in mortality from atherosclerotic heart disease has been observed in women over the last decade [8, 9].

Specific to CAD and CABG, whether these advances have resulted in unequivocal improvement in short-term morbidity and mortality for women has not been established.

Underrepresentation of women in early epidemiological studies has left significant voids in our understanding of female coronary artery disease and it is possible that gender-neutral advancements in care are benefiting men more than women. The aim of our study is to assess gender-differences in post-operative outcomes following CABG using hospital cardiac Society of Thoracic Surgeons (STS) surgical database for a gender-based analysis of 30-day outcomes following CABG surgery.

## Methods

This study is reported in accordance with the Strengthening the Reporting of Observational Studies in Epidemiology (STROBE) guidelines.

### Study design

We conducted a retrospective study using institutional data housed in the Society of Thoracic Surgeons (STS) database. The database is maintained using standardized collection tools by on-site, trained staff and certified surgical clinical reviewers (SCR) at participating institutions.

### Study setting & participants

Patients who underwent CABG surgery between 2002 and 2020 were included in the study. Patients younger than 18 years of age, and patients undergoing procedures in addition to CABG and revision surgery were excluded from the study. Additionally, patients with missing data for age (n = 56), sex (n = 26) and BMI (n = 44) were excluded from the study. Detail of missing variables is available in **S1 Table.**

### Variables

#### Independent variables

Data was electronically extracted for 130 relevant variables. The demographic variables of interest included age, gender, height, weight and body mass index (BMI). The data on patient comorbidities inclusive of diabetes, hypertension, smoking status, dialysis dependence and past history including prior MI, heart failure, arrhythmia, CABG or any other cardiac intervention (S2 Table) was extracted. Data on type of presentation to the hospital and ardiogenic shock at presentation was extracted. Data on preoperative use of ACE inhibitors, anti-platelets (including ADP inhibitors), heparin, beta blockers and baseline serum creatinine, hematocrit, ejection fraction, intra-aortic balloon pump was extracted. Procedure related variables included in the study were cross clamp, perfusion time, IMA utilization, number of diseased vessels and number of arterial and venous grafts.

#### Outcome variables

All post-operative outcomes were recorded till day of discharge from index admission. Readmission and mortality were recorded for a 30 day period after discharge. The primary outcomes for our study were post-operative outcomes including mortality, readmission due to cardiac related and non-cardiac related reasons, stroke, sepsis, deep sternal mediastinal infection, prolonged ventilator dependence, pneumonia, renal failure, heart block, cardiac arrest, GI complications, atrial fibrillation, re-operation for cardiac complications and re-operation for non-cardiac complications. Details of how outcome variables were derived from STS encoded variables are included in **S2 Table.**

### Quantitative variables and statistical analysis

Analysis was performed using SPSS Version 27. The primary end points were postoperative outcomes within 30 days following CABG including morbidity, length of stay, readmission and mortality. The Shapiro Wilk test for normalcy was used to distinguish between parametric and non-parametric variables. Based on the results, t-test was used for continuous variable and Mann-whitney U test was used for non-parametric variables to compare males and females. Chi square test was used for dichotomous variables. Multivariable logistic regression analysis was used to compare postoperative outcomes between males and females. The regression model adjusted for significantly different patient characteristics and additional variables as reported by previous literature and based on clinical plausibility. Threshold for significance for univariate was set at p<0.25. We applied Bonferroi correction to multivariable regression to reduce changes of type I error. The threshold for significance keeping the confidence interval at 95% (α = 0.05) was set at p<0.0045 for multivariable regression analysis. Linear regression analysis was performed for continuous variables to report standardized coefficients with coefficient standardized error.

#### Ethics

**The study was approved** the Institutional Review Board of Beth Israel Deaconess Medical Center and the need for informed consent was waived.

## Results

### Patient characteristics

**Fig 1:**
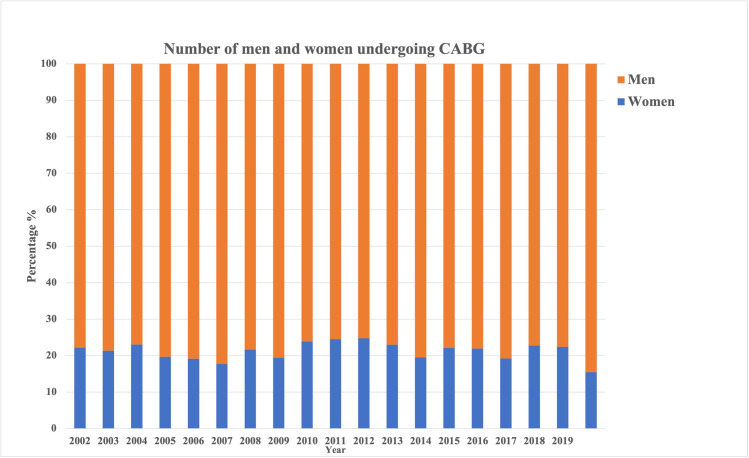
Number of men and women undergoing CABG surgery (2002–2019).

A total of 6,250 patients were included in the study of which 1,339 (21.4%) were women and 4,911 (78.6%) were men. The number of men and women presenting for CABG has not changed significantly over the years (p = 0.728, **Fig 1**). Our results show that women had a higher risk factor burden compared to men. 40.4% (n = 579) women were obese compared to 36.3% (n = 1,858) men (p-0.005). 52.0% (n = 745) women and 41.2% (n = 2,106) men were diabetic (p<0.001) and 89.1% (n = 1,278) women and 84.0% (n = 4,298) men were hypertensive (p<0.001). 50.7% (n = 727) women and 44.9% (n = 2,298) men had a history of previous myocardial infarction (p<0.001). 18.8% (n-269) women and 12.5% (n = 641) men had a history of heart failure (p<0.001). When compared to men, a larger proportion of women received anti-platelets (23.7%, n = 340 vs. 20.3%, n = 1,040, p = 0.006) and anti-coagulants (54.4%, n = 780 vs. 44.5%, n = 2,278, p<0.001) preoperatively. There was no difference in pre-operative prescription of ACE inhibitors and/or ARBs. (**Table 1**).

**Table 1:** Patient demographics and clinical characteristics stratified by sex.

Variable	Female (n = 1,339)		Male (n = 4,911)	*p-value*
**Demographics**				
Age*	67 (59–74)		66 (59–74)	0.447
Obesity (BMI > 30)	579 (40.4%)		1,858 (36.3%)	**0.005**
Smoking	627 (46.8%)		2,609 (53.1%)	**<0.001**
Diabetes	745 (52.0%)		2,106 (41.2%)	**<0.001**
Hypertension	1,278 (89.1%)		4,298 (84.0%)	**<0.001**
Dialysis dependence	17 (1.2%)		78 (1.5%)	0.342
History of cardiac Intervention	287 (20.0%)		1,002 (19.6%)	0.723
History of MI	727 (50.7%)		2,298 (44.9%)	**<0.001**
History of CABG	18 (1.3%)		100 (2.0%)	0.078
Heart failure	269 (18.8%)		641 (12.5%)	**<0.001**
History of arrythmia	172 (12.0%)		631 (12.3%)	0.760
**Preoperative variables**				
ACE Inhibitors	579 (40.5%)		1,958 (38.5%)	0.156
Anti-platelets	340 (23.7%)		1,040 (20.3%)	**0.006**
Heparin	780 (54.4%)		2,278 (44.5%)	**<0.001**
Beta Blockers	1274 (88.8%)		4487 (87.7%)	0.515
Creatinine (mg/dL)	1.07 ± 0.94		1.19 ± 0.99	**<0.001**
Ejection Fraction <45	358 (26.2%)		1,470 (30.5%)	**0.002**
Presentation				
No symptoms	36 (2.7%)		228 (4.8%)	**<0.001**
Stable angina	509 (37.9%)		2079 (43.6%)	
Unstable angina	478 (35.6%)		1502 (31.5%)	
NSTEMI	256 (19.1%)		773 (16.2%)	
STEMI	45 (3.3%)		132 (2.8%)	
Other	19 (1.4%)		56 (1.1%)	
Cardiogenic shock at presentation	11 (0.8%)		32 (0.7%)	0.606
Intra-aortic balloon pump (IABP)	96 (7.2%)		283 (5.8%)	**0.010**
**Intraoperative variables**				
Cross-clamp time (minutes)*		65 (52–80)	65 (51–79)	0.329
Bypass time (minutes)*		82 (67–99)	80 (66–96)	0.179
Number of diseased vessels				
One		56 (4.2%)	133 (2.7%)	**0.001**
Two		270 (20.2%)	853 (17.4%)	
Three		1010 (75.4%)	3922 (79.9%)	
Number of arterial grafts				
0		76 (5.7%)	126 (2.6%)	**<0.001**
1		1166 (87.1%)	4349 (88.6%)	
2		55 (4.1%)	261 (5.3%)	
3		33 (2.5%)	154 (3.1%)	
4		9 (0.6%)	9 (0.4%)	
5		0 (0%)	1 (0.02%)	
IMA utilization				
No IMA		119 (8.9%)	301 (6.1%)	**<0.001**
Left IMA		1160 (86.6%)	4334 (88.3%)	
Right IMA		6 (0.5%)	15 (0.3%)	
Bilateral IMA		54 (4.0%)	269 (5.3%)	
Number of venous grafts				
0		120 (9.0%)	399 (8.1%)	**<0.001**
1		233 (17.4%)	659 (13.4%)	
2		513 (38.3%)	1793 (36.5%)	
3		383 (28.6%)	1584 (32.3%)	
4		81 (6.0%)	428 (8.7%)	
5		9 (0.7%)	48 (1.0%)	

BMI: Body mass index; MI: myocardial infarction; CABG: Coronary artery bypass grafting; ACE: angiotensin converting enzyme; IMA: Internal mammary artery.

*Reported as median (Interquartile range).

### 30 day post-operative outcomes of CABG among male and female patients

A significantly larger proportion of women than men developed stroke (p<0.001), deep sternal mediastinal infection (p<0.001) prolonged ventilator dependence (p<0.001), renal failure (p<0.001), heart block (p<0.001) and cardiac arrest (p = 0.026). More women required reoperation for cardiac complications than men (p = 0.030). Both cardiac related (p<0.001) and non-cardiac related (p<0.001) readmission rates were significantly higher in women. Women also had a significantly longer duration of stay (p<0.001) higher rate of 30-day all-cause mortality (p<0.001) (**Table 2**).

**Table 2:** Outcomes of CABG stratified by sex and multivariable regression analysis showing odds of developing post-operative complications following CABG in female patients with males as reference.

Variable	Female (%)	Male (%)	p-value	Unadjusted analysis		Adjusted analysis^a^	
				OR	p-value	OR	p-value^b^
**Stroke**	46 (3.2)	54 (1.1)	**<0.001**				
**Sepsis**	10 (0.7)	24 (0.5)	0.288				
**DSMI**	14 (1.0)	12 (0.2)	**<0.001**				
**Ventilator dependence >48 hours**	200 (13.9)	398 (7.8)	**<0.001**	1.92 (1.60, 2.30)	<0.001	1.65 (1.21, 2.45)	**0.002**
**Pneumonia**	26 (1.8)	97 (1.9)	0.837				
**Renal Failure**	49 (3.4)	81 (1.6)	**<0.001**	2.20 (1.53, 3.15)	<0.001	0.99 (0.63, 1.54)	0.946
**Heart Block**	21 (1.5)	26 (0.5)	**<0.001**				
**Cardiac Arrest**	26 (1.8)	55 (1.1)	**0.026**				
**GI complications**	29 (2.0)	88 (1.7)	0.446	1.18 (0.77, 1.80)	0.447	0.94 (0.59, 1.49)	0.788
**Atrial Fibrillation**	338 (23.6)	1269 (24.8)	0.333	0.93 (0.81, 1.07)	0.333	1.10 (0.96, 1.26)	0.202
**Reoperation for cardiac complication**	17 (1.2)	32 (0.6)	**0.030**				
**Reoperation for non-cardiac complication**	66 (4.9)	191 (3.9)	0.094	1.37 (0.95, 1.98)	0.095	1.24 (0.23, 1.86)	0.293
**Cardiac readmission**	140 (9.8)	282 (5.5)	**<0.001**	1.85 (1.50, 2.29)	<0.001	1.56 (1.27, 2.30)	**0.003**
**Non-cardiac readmission**	161 (11.2)	324 (6.3)	**<0.001**	1.87 (1.53, 2.28)	<0.001	1.16 (0.77, 2.40)	0.402
**Duration of stay (days)** ^**c**^	7.19 ± 5.93	6.30 ± 4.64	**<0.001**	0.074 (0.148)	<0.001	-0.108 (0.182)	0.554
**30 day Mortality**	46 (3.2)	75 (1.5)	**<0.001**	2.23 (1.54, 3.23)	<0.001	1.55 (1.01, 2.40)	0.046

^a^Adjusted for, obesity, smoking status, diabetes, hypertension, history of myocardial infarction, heart failure, pre-operative ejection fraction and IABP use, heparin and anti-platelets, number of diseased vessels, IMA utilization, number of arterial and venous grafts and year of surgery.

^b^Bonferroni correction applied for multivariable regression—p<0.0045 considered significant.

^c^Results of Linear regression analysis reported as standardized coefficient (coefficient standardized error)

DSMI: Deep sternal mediastinal infection; GI: gastrointestinal; IMA: Internal mammary artery.

Multivariable regression analysis showed that women had significantly higher odds of developing prolonged ventilator dependence (p<0.001) and cardiac related readmission (p<0.001) than men after adjusting for confounding variables. After adjusting for confounding variables, odds of mortality in women were comparable to those of age matched men.

### Trends of morbidity and mortality

**Fig 2:**
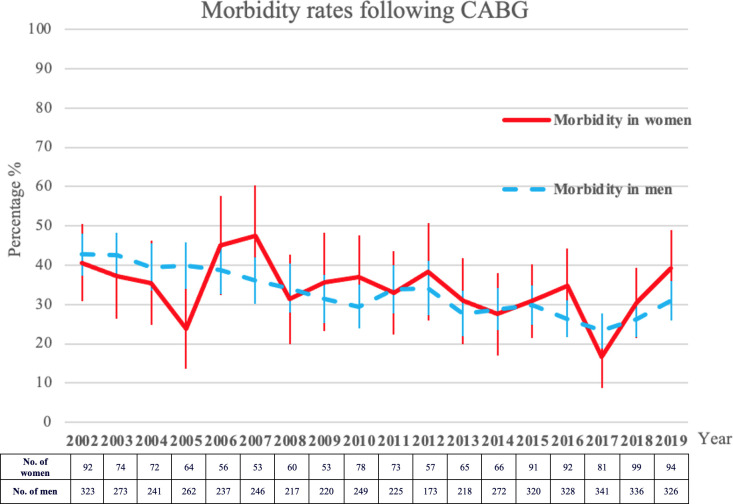
Mortality rates following CABG surgery in male and female patients.

**Fig 3:**
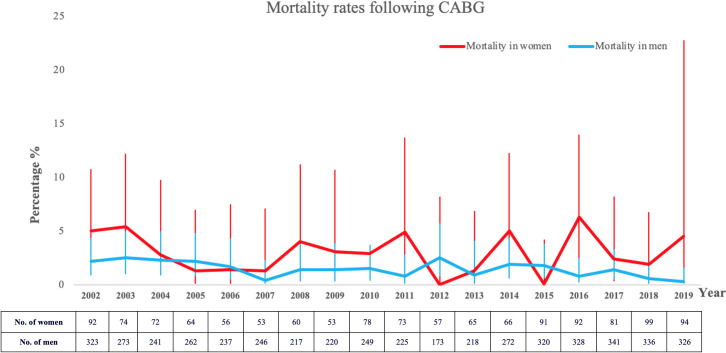
Morbidity (≥ 1 post-operative complication) rates following CABG in male and female patients.

Trends of morbidity and mortality are depicted in Figs 2 and 3.

## Discussion

Our study provides an update on gender-based differences in 30- day postoperative outcomes after CABG using most recent single center STS data. After adjusting for all potential confounding variables, women had comparable 30-day mortality with men; an improvement when compared to the mortality trends in past. Women had a higher incidence of prolonged ventilator dependence after adjusting for a higher comorbidity burden. The 30-day rate of cardiac readmission was also significantly higher in the women. The findings of our study indicate that despite apparent bridging of differences in all-cause 30-day mortality following CABG, the burden of postoperative complications and readmission rates is still high among women as compared to men.

Since the 1980s, mortality rates from CABG surgery have decreased from 45% to 2.2% with persistence of differences in mortality rates between men and women [10–13]. The unadjusted mortality rates differed significantly between men and women in our cohort, a finding consistent with more recent studies [10–12]. However, after adjusting for differences in demographic and clinical characteristics, the differences in mortality were eliminated and women did not have higher odds of mortality.

Gender and use of IMA graft has been identified as risk factors for sternal wound infections [14, 15] and the relationship is more pronounced in women. A study by Kieser et. al in 2014 reported that obese diabetic women undergoing bilateral internal mammary artery grafting had 10 fold higher risk of developing deep sternal wound infection than obese diabetic men [16]. Smaller vessel diameter, inadequate revascularization with higher chance of graft thrombosis, and underutilization of the IMA could possibly impact sternal healing and higher infection rate in women [17, 18].

Women are known to have a higher readmission rate than men (16% vs. 11% for 30 day and 32% vs. 21% for 6 months) [19, 20]. Besides unstable angina and heart failure as the major reasons, postoperative infections and pulmonary complications have also been implicated [19–21]. Prolonged ventilator dependence was significantly more common in women than men in our study. Whereas previous studies have shown varied reasons and equivocal findings [22, 23], the observed difference between genders in ventilatory requirements is not eliminated after adjustment for preoperative characteristics or other post-operative complications.

It appears that CAD often does not follow the typical pattern of obstructive CAD and women present later in the course of the disease possibly with increased post-menopausal disease pattern. Whereas previous studies have demonstrated that women with CAD are less likely to be the recipients of primary and secondary CAD prevention strategies [24–26], recent studies indicate that the gender-based disparities in delivery of evidence based care are reducing [27, 28]. Our study also shows that significantly more women than men were receiving preoperative anti-platelets and heparin and it is possible that we are headed towards a reversal rather than an elimination of sex-disparities in care.

More women than men were obese, diabetic, hypertensive and presented with prior histories of myocardial infarction and heart failure. Previous studies have also reported higher comorbidity burden in women presenting for CABG surgery and partially attribute differences in mortality to these observations [29].

Our analyses of data from approximately 6,500 patients explore in depth the outcomes of CABG in men and women after controlling for a range of pre, intra and post-operative confounders. Additionally, few recent studies have investigated gender differences in nonfatal and non-cardiac complications following CABG and report data up to 2016 (S3 Table). Since fewer women undergo CABG than men, the power of the study was higher for men than women due to a larger sample size. Additionally, data for some relevant variables including the use of radial artery as a conduit, degree of stenosis of vessels involved and the need for post-operative ventricular assist devices was not available.

## Conclusion

Women have higher rates of post-operative prolonged ventilator dependence and cardiac related readmission and mortality than men undergoing CABG surgery. Higher comorbidity burden is able to account for higher mortality rates in women but not for higher rates of said post-operative complications. The findings of our study indicate that despite apparent reduction of differences in all-cause short term mortality following CABG, the burden of postoperative complications and readmission rates is still high among women. Future efforts in primary prevention and research focused at understanding the molecular basis of observed differences between men and women is needed to reduce the gender disparity in CABG outcomes.

## Supporting information

S1 Table Missing variables. (DOCX)

S2 Table Derivation of outcome variables from STS encoded variables. (DOCX)

S3 Table Summary of findings from previous literature regarding gender differences in CABG outcomes. (DOCX)
