# Supplementary material for: Update: Gender differences in CABG outcomes—Have we bridged the gap?
Source: PLoS One. 2021 Sep 15;16(9):e0255170. doi: 10.1371/journal.pone.0255170 (PMC8443029; doi:10.1371/journal.pone.0255170)
Supplement: S1 Table — (DOCX) [file pone.0255170.s001.docx]

| **Variable** | **Missing number** |
| --- | --- |
| Age | 56 (excluded) |
| BMI | 44 (excluded) |
| Smoking | 32 |
| Diabetes | 7 |
| Hypertension | 13 |
| Dialysis dependence | 6 |
| History of cardiac  Intervention | 43 |
| History of MI | 25 |
| History of CABG | 18 |
| Heart failure | 57 |
| History of arrythmia | 61 |
| ACE Inhibitors | 30 |
| Anti-platelets | 2 |
| Heparin | 31 |
| Beta Blockers | 46 |
| Creatinine (mg/dL) | 12 |
| Ejection Fraction <45 | 362 |
| Presentation | 197 |
| Cardiogenic shock at presentation | 95 |
| Intra-aortic balloon pump | 922 |
| Cross-clamp time (minutes) | 312 |
| Bypass time (minutes) | 279 |
| Number of diseased vessels | 6 |
| Number of arterial grafts | 0 |
| IMA utilization | 0 |
| Number of venous grafts | 0 |
