# Supplementary material for: Update: Gender differences in CABG outcomes—Have we bridged the gap?
Source: PLoS One. 2021 Sep 15;16(9):e0255170. doi: 10.1371/journal.pone.0255170 (PMC8443029; doi:10.1371/journal.pone.0255170)
Supplement: S2 Table — (DOCX) [file pone.0255170.s002.docx]

**S2 Table. Derivation of outcome variables from STS encoded variables.**

| **Variable** | **Details** |
| --- | --- |
| **Gastrointestinal complications:** | - Ischemic Bowel - Gastrointestinal Bleed - Pancreatitis - Cholecystitis - Liver Dysfunction/Liver Failure - Ileus |
| **Cardiac readmission:** | - Pericardial effusion - MyocardiaI infarction - Angina - Coronary artery dysfunction - Congestive Heart Failure - Arrythmia/heart block - hypotension/hypertension |
| **Non-Cardiac readmission**: | - Wound (drainage, cellulitis) - TIA, Stroke - Respiratory complications/ Pneumonia/ Pleural effusion - Renal failure - PE - Deep sternal infection/ Conduit harvest site infection - GI Issues - Incisional complications - Electrolyte imbalance - DVT - Non cardiac chest pain - Anticoagulation complication |
| **Reoperation for cardiac complications:** | - Peri-operative MI - Reintervention for MI - Reoperation for graft occlusion - Reoperation for other cardiac related problems - Reoperation for valve dysfunction |
| **Re-operation for non-cardiac complications** | - As listed by STS - Re-operation for bleeding |
| **All other cardiac interventions:** | - Procedures for arrhythmias (ablation, Implantable Cardioverter Defibrillator, Permanent Pacemaker) - Aortic procedures - Ventricular assist device (VAD) - Pericardial window/Pericardiocentesis Pericardiectomy |
| **Morbidity** | - ≥1 post-operative complication or readmission |
