# Supplementary material for: Update: Gender differences in CABG outcomes—Have we bridged the gap?
Source: PLoS One. 2021 Sep 15;16(9):e0255170. doi: 10.1371/journal.pone.0255170 (PMC8443029; doi:10.1371/journal.pone.0255170)
Supplement: S3 Table — (DOCX) [file pone.0255170.s003.docx]

**S3 Table.** Summary of findings from previous literature regarding gender differences in CABG outcomes

| **Corresponding Author, year of publication** | **Sample** | **30-day Mortality, rates/Odds Ratio** | **30-day Readmission rates/Odds Ratio** | **30-day Morbidity rates/Odds Ratio** |
| --- | --- | --- | --- | --- |
| Walid Mohamed, **2020** | 2,537,767  (2004-2015) | Males: 2.1%  Females: 3.8%  Adjusted OR: 1.43 (1.40, 1.45), reference: male | - | Stroke  Males: 1.5%  Females: 3.4%  Adjusted OR: 1.34 (1.32, 1.37), reference: male |
| Amy Jhonston, **2019** | 52,546  (2008-2016) | Males: 1.2%  Females: 2.18% (p<0.001)  Adjusted OR: 1.15 (1.08, 1.21), reference: male | - | - |
| Soham Gupta, **2020** | 304,080  (2007-2014) | Overall: 1.87%  Males: 1.60%  Females: 2.65%  Adjusted OR: 1.32 (1.25-1.40), reference: male | Overall: 15.8%  Males: 14.3%  Females: 20.0% (p<0.001)  Adjusted OR: 1.24 (1.21, 1.28), reference: male | - |
| [Edmund R.Becker](https://www.sciencedirect.com/science/article/abs/pii/S0027968419300872#!), **2019** | 5,032,985  (1998-2015) | Overall: 2.2%  Male: 1.2%  Female: 3.2%  Females have a 43% higher chance of mortality | - | - |
| Francesco Nicolini, **2016** | 1,331  (2003-2016), propensity matched cohorts | Males: 1.3%  Females: 1.8%  (p=0.27) | Rates at 5-years  Males: 47.4%  Females: 45.9% (p=0.0499) | Stroke  Males: 0.1%  Females: 0.2% (p=0.561) |
| Rajesh V. Swaminathan, **2016** | 2,272,998  (2003-2012) | Males: 1.8%  Females: 3.2%  Adjusted OR: 1.40 (1.36, 1.43) (p<0.001), reference: male | - | Stroke  Males: 1.1%  Females: 1.7%  Adjusted OR: 1.35 (1.31, 1.40), (p<0.001), reference; male  Wound infection:  Males: 1.0%  Females: 1.3%  Adjusted OR: 1.15 (1.11, 1.18), (p<0.001), reference: male  Respiratory failure  Males: 7% Females: 9.1%  Adjusted OR: 1.21 (1.20, 1.23), (p<0.001), reference: male  Sepsis  Males: 1.2%  Females: 1.5%  Adjusted OR: 1.02 (0.99, 1.06), (p=0.16), reference: male |
| [Fraser D. Rubens](https://www-sciencedirect-com.ezp-prod1.hul.harvard.edu/science/article/pii/S0003497515017026#!), **2016** | 1,611  (2006-2014) | - | - | Sternal infection:  Adjusted OR: 3.964 (2.611, 6.018), (p<0.001) |
| Teresa M. Kieser, **2014** | 1001  (2003-2012) | Overall: 3.9% |  | Overall: 1.6% |
| David M. Shahian, **2014** | 162, 572  (2008-2010) | - | Overall: 16.8%  Males: 15.4%  Females: 20.1%  Adjusted OR: 1.38 (1.33–1.43) p<0.001, reference: male | - |
| [Jonathan D Price](https://pubmed.ncbi.nlm.nih.gov/?term=Price+JD&cauthor_id=23313544), **2013** | 1,205  (2006-2011) | - | Overall: 13%  Males: 12.1%  Female: 16.3% | - |
| [Zhongmin Li](https://pubmed.ncbi.nlm.nih.gov/?term=Li+Z&cauthor_id=22949489), **2012** | 11,283  (2009-2010) | - | Overall: 13.2%  Males: 12%  Females: 17.1%  Adjusted OR: 1.314 (p<0.001), reference: Male | - |
| [Kamran Mahmood](https://www.ncbi.nlm.nih.gov/pubmed/?term=Mahmood%20K%5BAuthor%5D&cauthor=true&cauthor_uid=22617003), **2012** | 261,255  (2004-2008) | Males: 11.8%  Females: 12.9% ( p<0.001)  Adjusted OR: 0.98 (0.95, 1.02), (p=0.39), reference: male |  |  |
| Akshat Saxena, **2011** | 21,534  (2001-2009) | Males: 1.5%  Females: 2.2% (p<0.001)  Adjusted OR:  0.93 (0.68, 1.27), (p= 0.638) |  | Permanent stroke:  Males: 0.71% Females: 1.05 % (p=0.681)  Transient stroke: Males: 0.44%  Females: 0.32% (p=0.098)  Sternal wound infection: Males: 0.77%  Females: 0.61% (p=0.017) |
| Viola Vaccarino, **2003** | 1,072  (1999-2001) | - | Rates at 6 months  Males: 21.2%  Females: 32.6%  Adjusted OR: 1.53 (p=0.01), reference: male | - |
| [Constance K.Haan](https://www-sciencedirect-com.ezp-prod1.hul.harvard.edu/science/article/pii/S0002914903002789#!), **2003** | 129,710  (1997-2000) | Males: 5.2%  Females: 6.5%  Adjusted OR: 0.882 (0.823, 0.944), reference: female |  | Stroke  Males: 2.8%  Females: 3.31%  Adjusted OR: 0.931 (0.849, 1.021), reference: female  Prolonged ventilation Males: 8.6% Females: 10.6% Adjusted OR: 0.797 (0.754, 0.843), reference: female  Deep sternal infection:  Males: 0.70% Females: 0.50%  Adjusted OR: 0.876 (0.727, 1.055), reference: female |
| Viola Vaccarino, **2002** | 51,187  (1993-1999) | Males: 2.9%  Females: 5.3%  Adjusted OR: 1.16 (0.93, 1.45), reference: male | - | Stroke/TIA Males: 3.8%  Females: 5.3%  Adjusted OR: 1.6 (p<0.001), reference: male |
| LC Hussey, **2001** | 306^c^  (1989-1999) |  |  | Sternal infection:  Males: 39.3%  Females: 32.5% |
| R D Stewart, **2000** | 485  (1997) | - | Overall: 16%  Males: 11%  Females: 25%  Adjusted OR: 2.145 (1.44-4.20) p<0.001, reference: male | - |
